# Evaluation of the Fecal Bacterial Communities of Angus Steers With Divergent Feed Efficiencies Across the Lifespan From Weaning to Slaughter

**DOI:** 10.3389/fvets.2021.597405

**Published:** 2021-06-29

**Authors:** Christina B. Welch, Jeferson M. Lourenco, Taylor R. Krause, Darren S. Seidel, Francis L. Fluharty, T. Dean Pringle, Todd R. Callaway

**Affiliations:** Department of Animal and Dairy Science, University of Georgia, Athens, GA, United States

**Keywords:** beef cattle, *Bifidobacteriaceae*, *Christensenellaceae*, fecal microbiome, residual feed intake, *Rikenellaceae*, *Ruminococcaceae*, productive life

## Abstract

Numerous studies have examined the link between the presence of specific gastrointestinal bacteria and the feed efficiency of cattle. However, cattle undergo dietary changes during their productive life which can cause fluctuations in their microbial consortium. The objective of the present study was to assess changes in the fecal microbiome of beef steers genetically selected to be divergent in feedlot feed efficiency, to determine whether differences in their fecal microbiomes could be detected as early as weaning, and continued throughout the rearing process regardless of dietary changes. Fecal samples were collected at weaning, yearling age, and slaughter for a group of 63 steers. Based on their feedlot-finishing performance, the steers were selected and divided into two groups according to their residual feed intake (**RFI**): efficient steers (low-RFI; *n* = 7) and inefficient steers (high-RFI; *n* = 8). To ascertain the fecal microbial consortium and volatile fatty acid (VFA) content, 16S rRNA gene sequencing and VFA analysis were performed. Overall, bacterial evenness and diversity were greater at weaning compared to yearling and slaughter for both efficiency groups (*P* < 0.001). Feedlot RFI linearly decreased as both Shannon diversity and *Ruminococcaceae* abundance increased (*R*^2^ = 65.6 and 60.7%, respectively). Abundances of *Ruminococcaceae, Rikenellaceae*, and *Christensenellaceae* were higher at weaning vs. yearling age and slaughter (*P* < 0.001); moreover, these families were consistently more abundant in the feces of the low-RFI steers (for most of the timepoints evaluated; *P* ≤ 0.05), compared to the high-RFI steers. Conversely, abundances of *Bifidobacteriaceae* were numerically higher in the feces of the high-RFI steers throughout their lifespan. Total VFA concentrations increased at slaughter compared to weaning and yearling for both efficiency groups (*P* < 0.001). The acetate:propionate ratio decreased linearly (*P* < 0.001) throughout the life of the steers regardless of their efficiency, reflective of dietary changes. Our results indicate that despite fluctuations due to animal age and dietary changes, specific bacterial families may be correlated with feed efficiency of steers. Furthermore, such differences may be identifiable at earlier stages of the production cycle, potentially as early as weaning.

## Introduction

In beef production systems, feed represents the largest single cost and accounts for an estimated 60–75% of the total cost of production (1). In order to increase the profitability of beef operations, producers seek to improve the efficiency by which cattle convert ingested feed into body weight gain (2, 3). One method to determine the feed efficiency of cattle is to calculate their residual feed intake (RFI). Concisely, RFI is the difference between the observed and the expected feed intake, based on metabolic body weight and a certain level of gain. If an animal eats less than expected for that level of gain (low-RFI), it is considered more efficient (3, 4). Therefore, low-RFI animals are more efficient than animals that have high-RFI values.

An estimated 19% of the variation in RFI can be attributed to diet composition and digestibility of feed (5). Variation in RFI can also be linked to the microbial population within the gastrointestinal tract (**GIT**) of cattle because microbes produce 70% of the energy and 50% of the protein the ruminant animal uses (6, 7). Many studies have found a direct link between feed efficiency and the microbial population in cattle's GIT (8–10); however, most of these studies targeted on the ruminal microbial population. In contrast, a recent study has shown that certain bacterial families present in the hindgut of cattle can be correlated with feedlot RFI and may be important in driving the host's feed efficiency (11); however, it is still unknown at what point these bacterial families diverge within the hindgut of steers.

The present study was designed to evaluate the composition of the fecal microbial population of beef cattle that differed greatly in feed efficiency (as assessed by feedlot RFI). In practical beef production, obtaining fecal samples is substantially easier than taking ruminal samples. Therefore, fecal samples were collected from steers at three different times during their production cycle: at weaning, yearling age, and immediately post-slaughter. The fecal microbiome of the most efficient (low-RFI) and least efficient (high-RFI) steers were evaluated and compared at each stage. We hypothesized that fecal microbiomes would consistently differ at different points in the lifecycle of steers based on their feed efficiency evaluated during the feedlot-finishing phase, and that the fecal microbiomes would differ within those timepoints based on efficiency group.

## Materials and Methods

### Animals, Diets, and Steer Selection

The steers utilized in this study were cared for using the guidelines approved by the University of Georgia's Animal Care and Use Committee (AUP #A2012 11-006-R1). The steers used in the present study are from the fifth generation of a genetic selection program involving Angus cattle being selected for residual average daily gain and intramuscular fat (marbling). All steers were born (12/7/2016–1/22/2017) and raised at the Northwest Georgia Research and Education Center, located in Calhoun, GA (34° 30 N, 84° 57 W) where they were reared in a pasture-based system until ~10 months of age. The steers (*n* = 63) were then transported to a commercial feedlot located in Brasstown, NC (35° 10 N, 83° 23 W) where they were backgrounded prior to starting the feedlot trial. During the feedlot trial, the steers were maintained under a GAP 4 certification utilizing a non-hormone treated cattle program. Prior to the start of the finishing phase, steers were accustomed to high-grain diets over the course of 3 weeks. The finishing period lasted 110 days. The finishing diet contained 14.51% crude protein, 2.10 Mcal/kg NE_m_, 1.43 Mcal/kg NE_g_, 0.70% Ca, and 0.45% P on a DM basis. Further composition information on both the transition and finishing diets can be found in Supplementary Table 1. Additionally, body weight was recorded at birth, weaning, yearling age (start of the feedlot period), and the conclusion of the feedlot trial.

During the feedlot period, the feed intake of steers was individually measured using a GrowSafe System (GrowSafe Systems Ltd., Calgary, Canada). Intake data was then used to calculate their individual feed conversion rates, which were expressed as RFI. Feed intake data was also used to calculate the daily cost of feeding the steers. Upon conclusion of the feedlot period, steers were rank-ordered based on their feed efficiencies (i.e., RFI) and the 12 most efficient (lowest RFI values), along with the 12 least efficient (highest RFI values) were transported to the University of Georgia Meat Science Technology Center, a federally inspected meat plant located in Athens, GA (33° 57 N, 83° 22 W). The steers were housed on site overnight where they were fasted but given *ad libitum* access to water prior to slaughter the next morning. In order to create an even greater biological distinction between the two groups of steers and have a greater difference in the magnitude of their RFI values, further selection of the samples was performed, resulting in a total of 15 steers being used in this study: 7 classified as low-RFI (efficient steers), and 8 classified as high-RFI (inefficient steers).

### Fecal Collection and Storage

The first set of fecal samples was collected at weaning (~9 months of age) on all steers (*n* = 63). Fecal contents were aseptically collected via fecal grab (~50 g) using a separate palpation sleeve for each sample. The feces were then placed in 50 mL conical tubes and stored on ice until the samples were transferred to the laboratory and stored at −20°C. After the steers were transported to the feedlot, backgrounded, and started on the finishing diet, the second set of fecal samples were collected on all steers (*n* = 63), which corresponded to the yearling phase (~13 months of age). These samples were collected and prepared as described above. The final set of samples was collected from their rectum upon evisceration of the carcasses on slaughter day (~18 months of age) as mentioned above, and immediately placed in a −20°C freezer for storage.

### DNA Extraction and Sequencing

Microbial DNA was extracted from the feces of the 7 less-efficient and the 8 most-efficient steers using a hybrid DNA extraction protocol utilizing both mechanical and enzymatic methods as previously described by Rothrock et al. (12). This procedure uses 0.33 g of fecal material placed into 2-mL Lysing Matrix E tubes (MP Biomedicals LLC, Irvine, CA, USA) which are homogenized using a FastPrep 24 Instrument (MP Biomedical LLC, Irvine, CA, USA) to mechanically break open the cells. InhibitEX Tablets (QIAGEN, Venlo, Netherlands) were used as the enzymatic means of increasing DNA yields. An automated robotic workstation (QIAcube; QIAGEN, Venlo, Netherlands) was used for elution and purification of DNA from the samples. DNA concentration and purity were determined spectrophotometrically using the Synergy H4 Hybrid Multi-Mode Microplate Reader along with the Take3 Micro-Volume Plate (BioTek Instruments Inc.; Winooski, VT, USA). The samples required at least 20 μL of volume and a concentration of 10 ng/μL of DNA in order to proceed to sequencing. Samples that failed to meet these minimum requirements were processed through a new cycle of DNA extraction. Once all samples were adequate in both volume and DNA concentration, they were stored at 4°C overnight.

After overnight storage, the samples were transported to the Georgia Genomics and Bioinformatics Core (https://dna.uga.edu) for library preparation and 16S rRNA gene sequencing. The library preparation included PCR replications using the forward primer: S-D-Bact-0341-b-S-17 (5′-CCTACGGGNGGCWGCAG-3′) and reverse primer: S-D-Bact-0785-a-A-21 (5′-GACTACHVGGGTATCTAATCC-3′) (13, 14). PCR conditions were: initial denaturation at 95°C for 3 min, followed by 25 cycles of 95°C for 30 s, annealing at 55°C for 30 s, extension at 72°C for 30 s, and then a final elongation step at 72°C for 5 min. PCR clean-up was performed using AMPure XP beads (Beckman Coulter Life Sciences, Indianapolis, IN, USA). The library was quantified using qPCR, and the V3-V4 variable regions of the 16S rRNA gene were sequenced using an Illumina MiSeq instrument with a MiSeq v3 reagent kit for lengths of 2 × 300 bp (Illumina Inc., San Diego, CA, USA). A well-characterized bacteriophage PhiX genome (PhiX Control v3 Library; Illumina Inc., San Diego, CA, USA) was used as a control for the sequencing runs.

### Sequencing Data

After sequencing was performed, the data was demultiplexed and converted into FASTQ files. Pair-end reads were set and merged using BBMerge Paired Read Merger v37.64 with an expected insert size of 500 bp, and files were analyzed using QIIME pipeline v1.9.1 (15). The files were then filtered based on quality (minimum Phred quality score of 20) and merged into one single file that was converted into the FASTA format. Sequences were grouped together at 97% similarity into operational taxonomic units (OTU) using the Uclust method and the Greengenes database (gg_13_8_otus). Sequence depth was set at 17,542 sequences per sample for further analysis. This value was selected because it allowed the retention of all the samples while providing a minimum Good's coverage index of 0.95. The data was made publicly available, and readers can find it at: https://www.mg-rast.org using the accession number: mgm4909317.3. Phylogenetic Investigation of Communities by Reconstruction of Unobserved States (PICRUSt) was performed to make inferences about the metabolic pathways expressed within the microbiota (16, 17); and the metabolic functions were assessed using the Kyoto Encyclopedia of Genes and Genomes (KEGG) third-level pathways.

### Volatile Fatty Acid Analysis

Analysis of volatile fatty acids (**VFA**) was performed according to the procedure described in Lourenco et al. (18). One gram of feces was diluted with 3 mL of distilled water and placed into 15-mL conical tubes. The tubes were vortexed for 30 s to produce a homogeneous sample and 1.5 mL of the mixture was transferred to microcentrifuge tubes. The tubes were centrifuged at room temperature at 10,000 × *g* for 10 min. One milliliter of the supernatant was transferred into a new microcentrifuge tube and mixed with 0.2 mL of metaphosphoric acid solution (25% v/v). The samples were vortexed for 30 s and stored at −20°C overnight. The next morning, samples were thawed and centrifuged at room temperature at 10,000 × *g* for 10 min. The supernatant was removed and transferred into polypropylene tubes combined with ethyl acetate in a 2:1 ratio of ethyl acetate to supernatant. Tubes were vortexed for 10 s to thoroughly mix them and allowed to settle for 5 min for optimum separation. Then 600 μL of the top layer was transferred into screw-thread vials. VFA analysis was performed using a Shimadzu GC-2010 Plus gas chromatograph (Shimadzu Corporation, Kyoto, Japan) with a flame ionization detector and a capillary column (Zebron ZB-FFAP; 30 m × 0.32 mm × 0.25 μm; Phenomenex Inx., Torrance, CA, USA). Sample injection volume was set to 1.0 μL, and helium was used as a carrier gas. Column temperature started at 110°C and increased to 200°C over the course of 6 min. The injector temperature was set to 250°C, and the detector temperature was set to 350°C.

### Statistical Analysis

**Figure 1:**
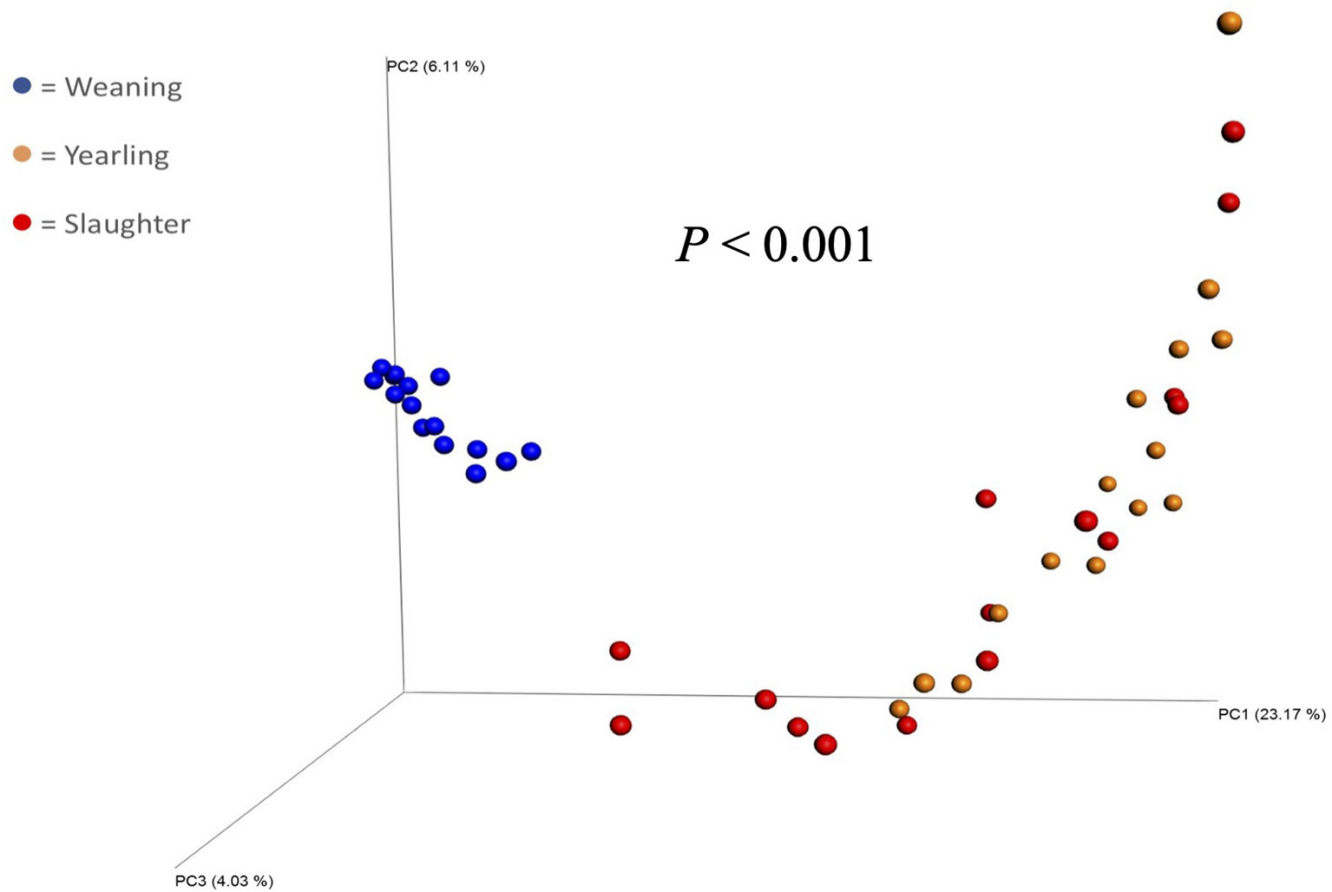
Principal coordinate analysis plot of beta diversity (unweighted UniFrac) of fecal bacterial populations of steers (*n* = 15) collected at weaning, yearling, and slaughter. *P*-value indicates a difference in beta diversity between samples collected at each timepoint.

Statistical analyses were performed using Minitab v19.1. Animal performance data [birth weight, weaning weight, yearling age weight, weight at the end of the feedlot period, feedlot dry matter intake, feedlot feed costs, feedlot feed:gain, and feedlot residual feed intake], alpha-diversity indices, and bacterial abundances were analyzed using a one-way ANOVA with feedlot RFI classification (i.e., high- or low-RFI) as a factor. In addition, repeated-measures ANOVA were carried out for each group of steers to investigate potential differences across the 3 samples collected throughout their lifecycle (weaning, yearling, and slaughter), and Tukey's pairwise comparisons were performed to assess further differences. Multiple correlations between RFI values and the microbial traits were evaluated, and both Shannon diversity and the abundance of *Ruminococcaceae* at slaughter were found to be highly significant. Thus, linear regression analysis was performed to investigate the relationship between Shannon diversity index at slaughter and RFI; as well as between the abundance of *Ruminococcaceae* at slaughter and RFI. Multiple correlations were performed between bacterial abundance and the expression of metabolic pathways (Supplementary Table 2). Given that the abundance of *Rikenellaceae* was positively associated with glycosaminoglycan degradation, a linear regression was performed between these two traits in the feces of steers across all stages of production. Anderson-Darling Normality Tests were performed on the alpha diversity metrices and the bacterial abundances at each time point for both efficiency groups, and the majority were normally distributed (Supplementary Figures 1–6). Beta diversity between all pairs of samples was calculated using QIIME's “beta_diversity_through_plots.py” script and results were visualized using 3-dimensional plots (Figure 1). Unweighted UniFrac distances were used for the beta diversity plots. This metric was chosen because it accounts for phylogenetic relationships when measuring beta diversity (19). For all statistical tests, results were considered significant at *P* ≤ 0.05, and treated as trends when 0.05 < *P* ≤ 0.10.

## Results

### Animal Performance

Body weight was consistent throughout the life cycle of the efficient and the inefficient steers (Table 1; *P* ≥ 0.51). Dry matter intake during the feedlot-finishing period was lower for the low-RFI (efficient) steers (*P* < 0.001) compared to the high-RFI (inefficient) steers, resulting in a decrease in the daily feeding cost for the efficient steers (*P* < 0.001) compared to the inefficient steers. Feed conversion, expressed as a feed:gain ratio, was lower (*P* = 0.001) for the more efficient steers. Likewise, the efficient and inefficient steers were divergent (*P* < 0.001) in terms of feedlot RFI, with the efficient steers consuming 4.04 kg less dry matter per day when comparing to the inefficient steers (RFI = 2.02 and −2.02 for the inefficient and efficient steers, respectively).

**Table 1:** Performance of the efficient and inefficient steers (*n* = 7 efficient and *n* = 8 inefficient) at different points in the beef production continuum.

**Performance trait**	**Inefficient**	**Efficient**	**SEM**	***P*-value**
Birthweight, kg	38.4	37.2	1.3	0.52
Weaning (9 months-old) weight, kg	300	298	10.5	0.90
Yearling weight (13 months-old), kg	501	487	14.4	0.51
Feedlot final body weight (18 months-old), kg	599	584	15.6	0.51
Feedlot dry matter intake, kg/d	13.6	9.7	0.87	<0.001
Feedlot daily feeding cost, US$/steer^*^	3.13	2.22	0.14	<0.001
Feedlot feed:gain ratio, kg	15.84	10.54	1.32	0.001
Feedlot residual feed intake (RFI), kg/d	2.02	−2.02	0.82	<0.001

* *The feedlot-finishing diet had a cost of US$ 0.23/kg DM*.

### Diversity Indices

**Figure 2:**
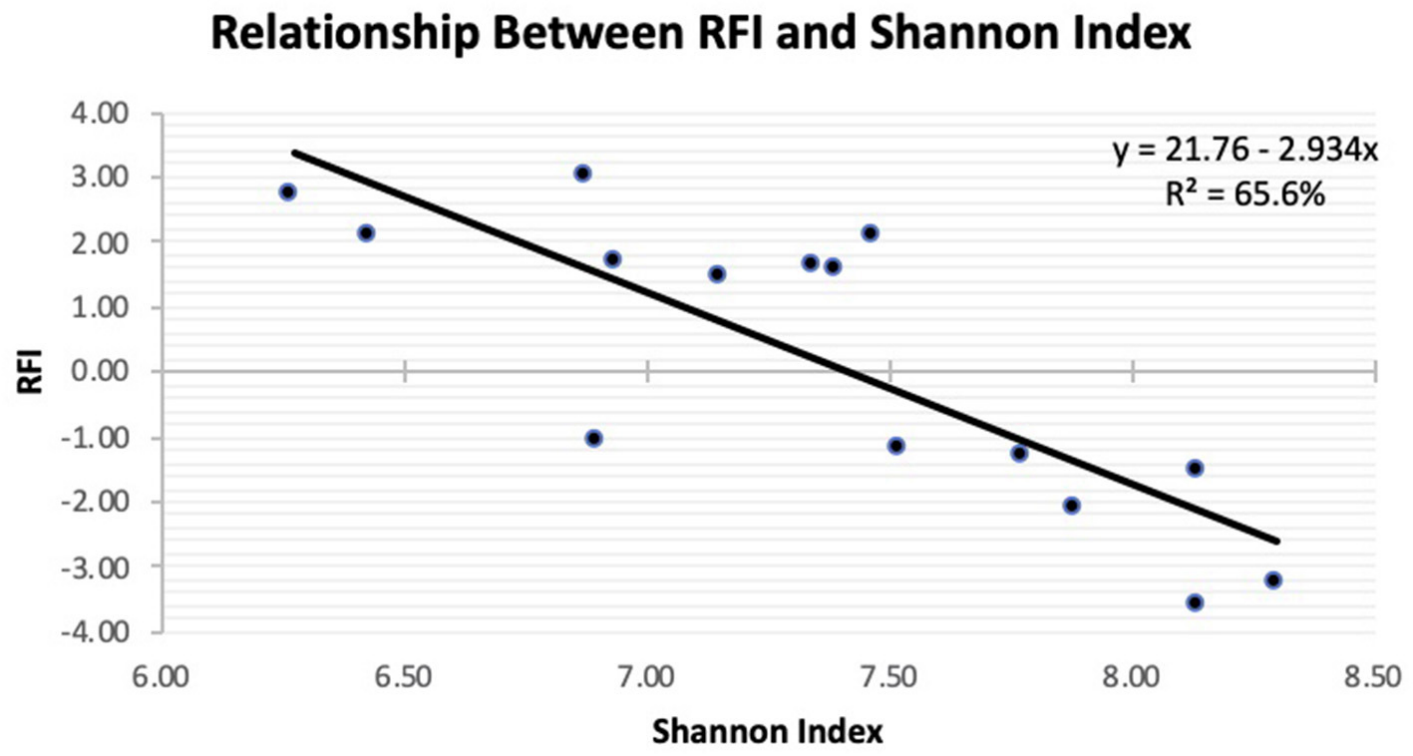
Linear regression expressing the relationship between residual feed intake (RFI) and Shannon diversity in the feces of Angus steers observed at slaughter.

The Principal Coordinate Analysis (PCoA) plot of Beta-diversity for the steers at weaning, yearling, and slaughter showed that fecal samples collected at weaning were different (*P* < 0.001); whereas, fecal samples collected at yearling and slaughter clustered together (Figure 1). There was no difference (*P* = 0.080) in beta diversity between efficiency group (Supplementary Figure 7). Alpha-diversity indices for the efficient and inefficient steers at weaning, yearling, and slaughter were examined (Table 2). In the inefficient steers, Chao 1, an indicator of microbial richness, was higher (*P* = 0.02) in the feces at weaning compared to the feces collected at slaughter. In the efficient steers, microbial richness at slaughter and yearling numerically decreased compared to weaning, but this was not significant (*P* = 0.19). In both the efficient and inefficient steers, the species within the feces at weaning were more evenly distributed (*P* < 0.001) than at yearling and slaughter. Similarly, the Shannon diversity index of fecal samples was higher (*P* < 0.001) at weaning than at the later timepoints, regardless of feedlot efficiency status of the steers. Regression analysis revealed that as the Shannon diversity index increased, RFI decreased (Figure 2; *R*^2^ = 65.6%). The Shannon diversity index did not differ between efficient and inefficient steers at weaning and yearling (Table 2; *P* ≥ 0.18); however, at slaughter, fecal microbial diversity was greater (*P* = 0.004) in the efficient steers compared to inefficient steers. Chao 1 index did not differ between inefficient and efficient steers at any timepoint (*P* ≥ 0.13). Microbial evenness did not differ between inefficient and efficient steers at weaning or yearling (*P* ≥ 0.15) but was greater (*P* = 0.001) in the efficient steers compared to the inefficient steers at slaughter.

**Table 2:** Alpha-diversity indices calculated for the fecal samples of efficient and inefficient steers at different stages of their lives: weaning, yearling, and kill floor (slaughter).

**Index**		**Weaning**	**Yearling**	**Slaughter**	**SEM**	***P*-value**
Chao1	Inefficient	4360.6^a^	3956.9^ab^	3384.7^b^	206	0.02
	Efficient	4424.8	3621.4	3834.8	303	0.19
	SEM	178	109	205		
	*P*-value	0.86	0.13	0.29		
Species evenness	Inefficient	0.788^a^	0.687^b^	0.658^b^	0.009	<0.001
	Efficient	0.804^a^	0.710^b^	0.724^b^	0.006	<0.001
	SEM	0.006	0.009	0.012		
	*P*-value	0.15	0.20	0.001		
Shannon diversity	Inefficient	8.73^a^	7.42^b^	6.99^b^	0.14	<0.001
	Efficient	8.95^a^	7.61^b^	7.81^b^	0.11	<0.001
	SEM	0.080	0.100	0.159		
	*P*-value	0.18	0.36	0.004		

ab *Values not sharing a common superscript within each row significantly differ according to Tukey's pairwise comparison (P ≤ 0.05)*.

### Bacterial Relative Abundance

**Figure 3:**
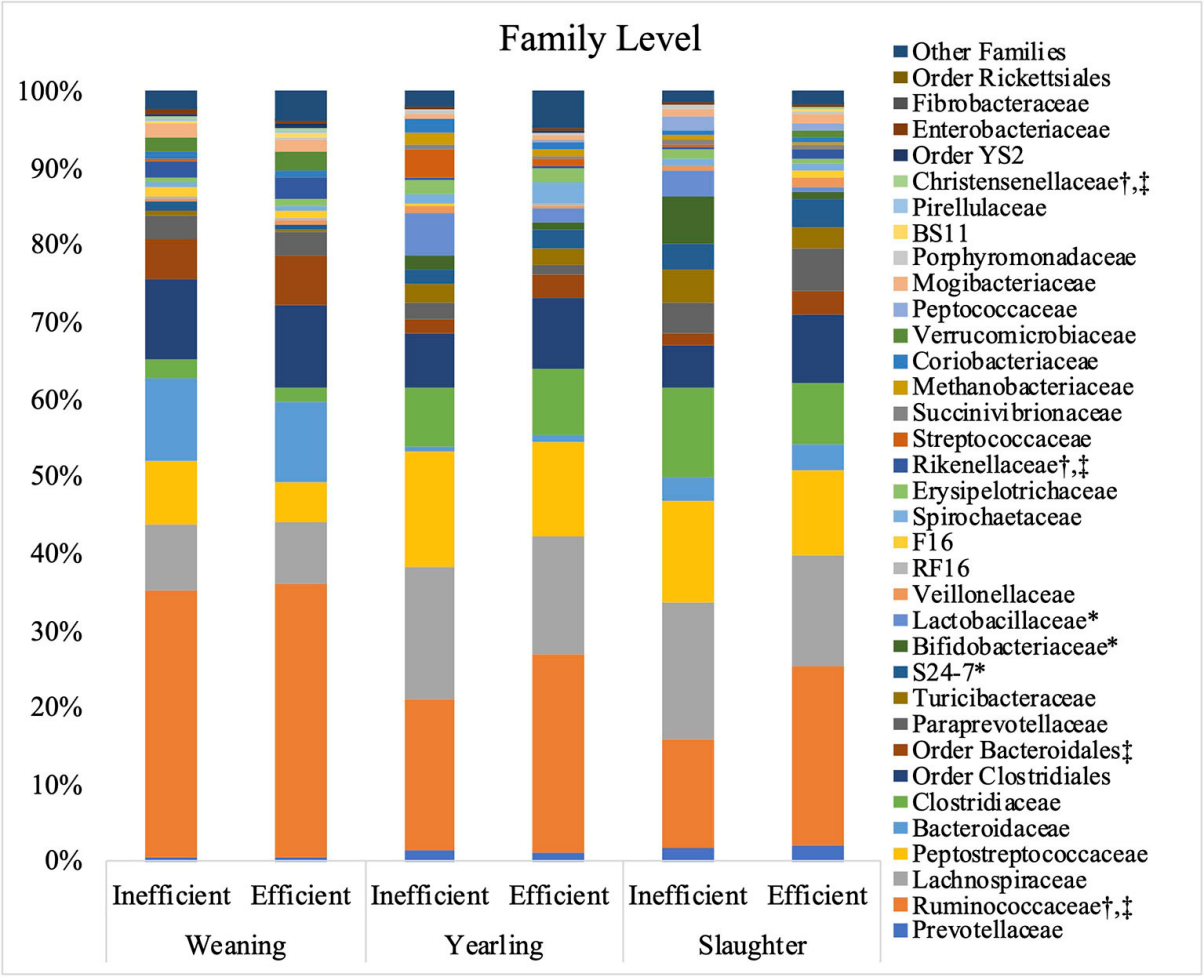
Relative bacterial abundance at the family level found in the feces of efficient and inefficient steers at weaning, yearling, and slaughter. Differences between the two groups of steers (*P* ≤ 0.05) are denoted by * ^†^ at weaning, at yearling, and ^‡^at slaughter.

The relative abundances of the bacterial families S24-7, *Bifidobacteriaceae*, and *Lactobacillaceae* were greater (*P* ≤ 0.05) in the inefficient steers at weaning compared to the efficient steers (Figure 3). At both yearling and slaughter, abundance of the families *Ruminococcaceae, Rikenellaceae*, and *Christensenellaceae* were higher (*P* ≤ 0.05) in feces of efficient steers than in feces of the inefficient steers.

**Figure 4:**
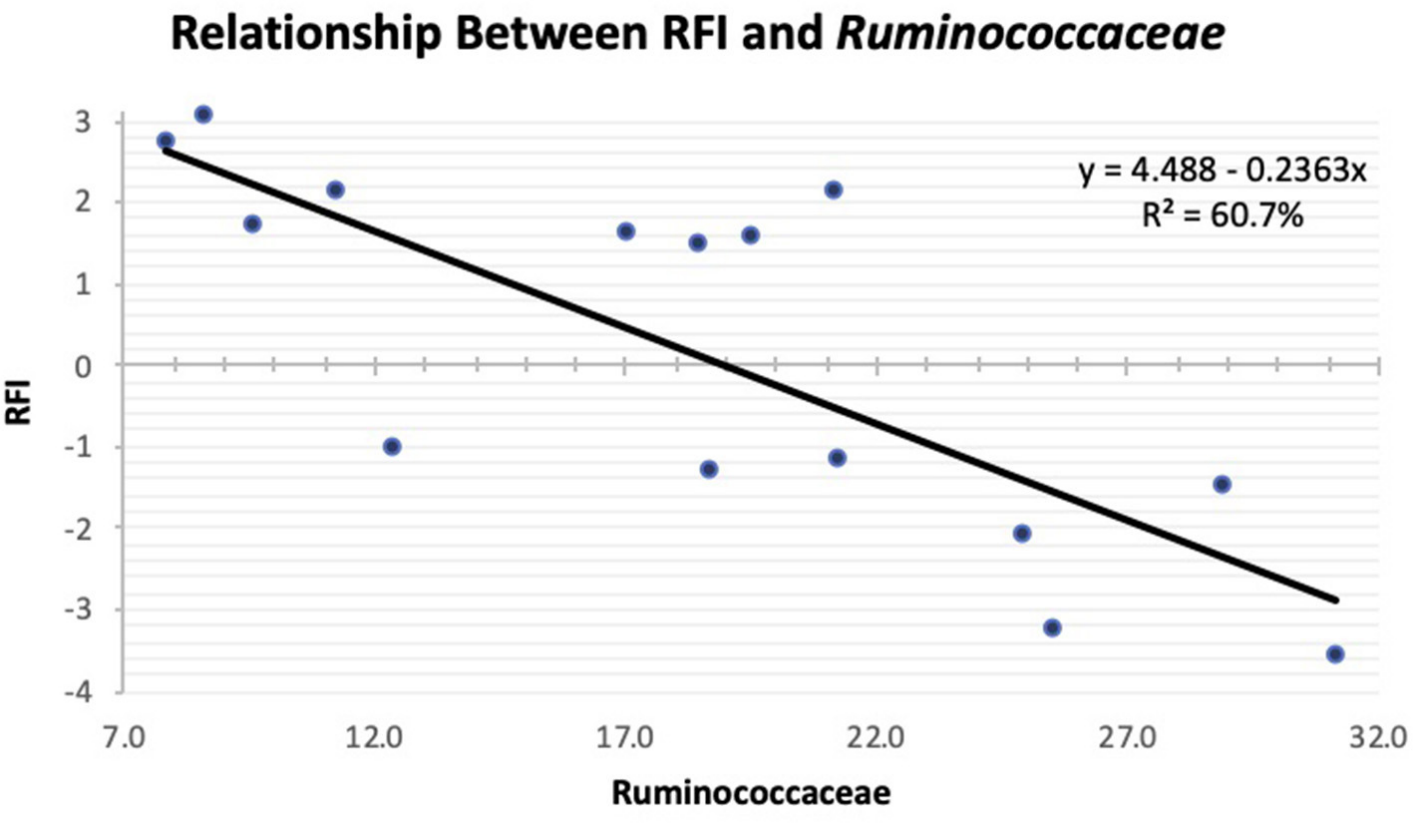
Linear regression expressing the relationship between RFI and abundance of *Ruminococcaceae* in the feces of Angus steers at slaughter.

**Figure 5:**
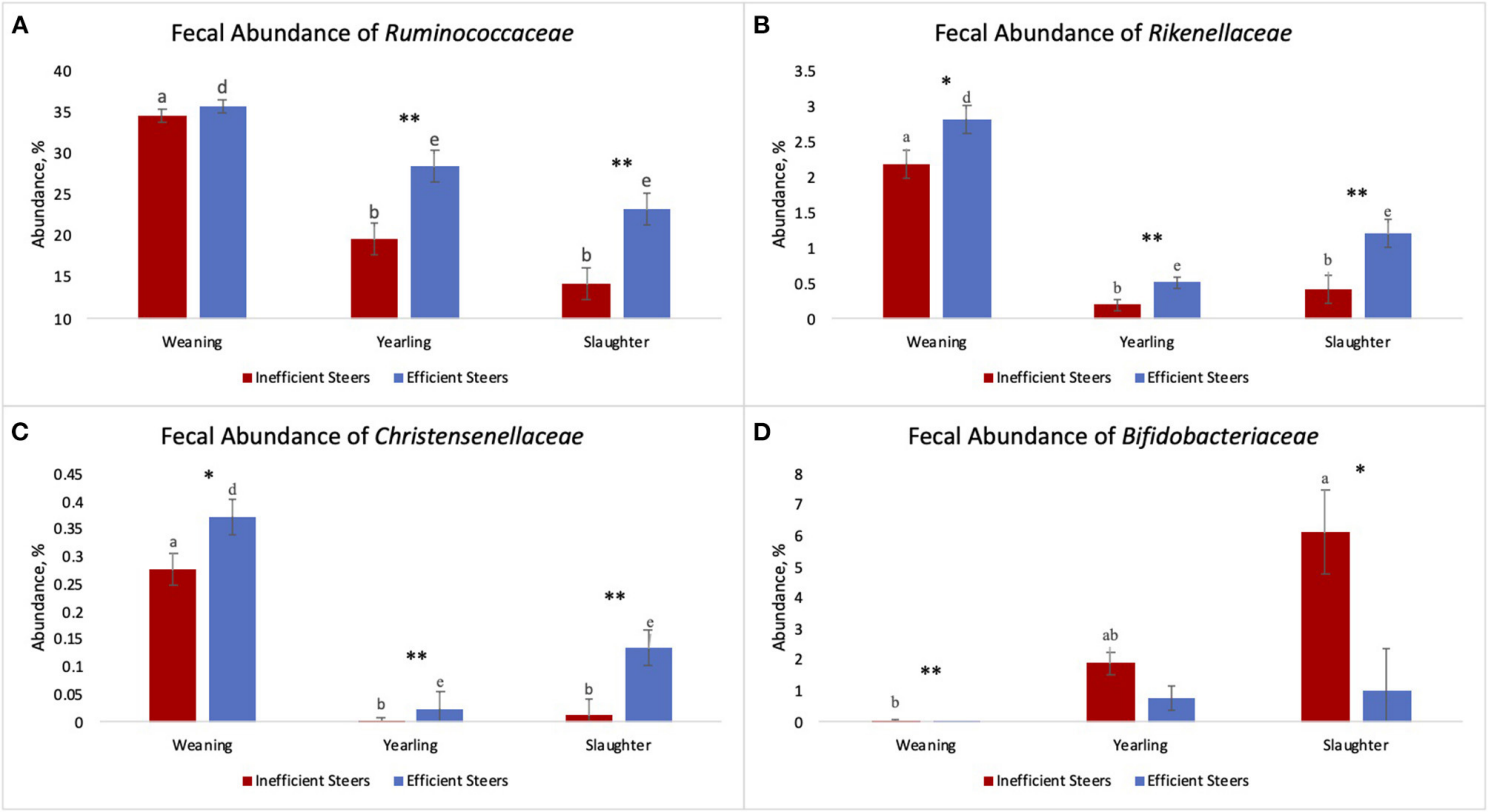
Fecal abundance of *Ruminococcaceae* **(A)**, *Rikenellaceae* **(B)**, *Christensenellaceae* **(C)**, and *Bifidobacteriaceae* **(D)** at weaning, yearling, and slaughter of inefficient (*n* = 8) and efficient (*n* = 7) steers. ^a,b,c^ indicate a significant difference across timepoints (*P* ≤ 0.05) of inefficient steers. ^d,e,f^ indicate a significant difference across timepoints (*P* ≤ 0.05) of efficient steers. Asterisks indicate a difference between inefficient and efficient steers at individual timepoint with * indicating a trend (0.10 ≤ *P* ≥ 0.05) and ** indicating a significant difference (*P* ≤ 0.05). Error bars indicate standard error.

As the population of *Ruminococcaceae* increased in the feces collected at slaughter, the RFI of the host was lower (Figure 4; *R*^2^ = 60.7%). *Ruminococcaceae* abundance was higher (*P* < 0.001) at weaning than at both yearling and slaughter in both feed efficiency groups (Figure 5A). At weaning, there were no differences (*P* = 0.46) in *Ruminococcaceae* abundance between efficient and inefficient steers. At yearling and slaughter, *Ruminococcaceae* abundance was higher (*P* = 0.01) in efficient steers compared to inefficient steers.

**Figure 6:**
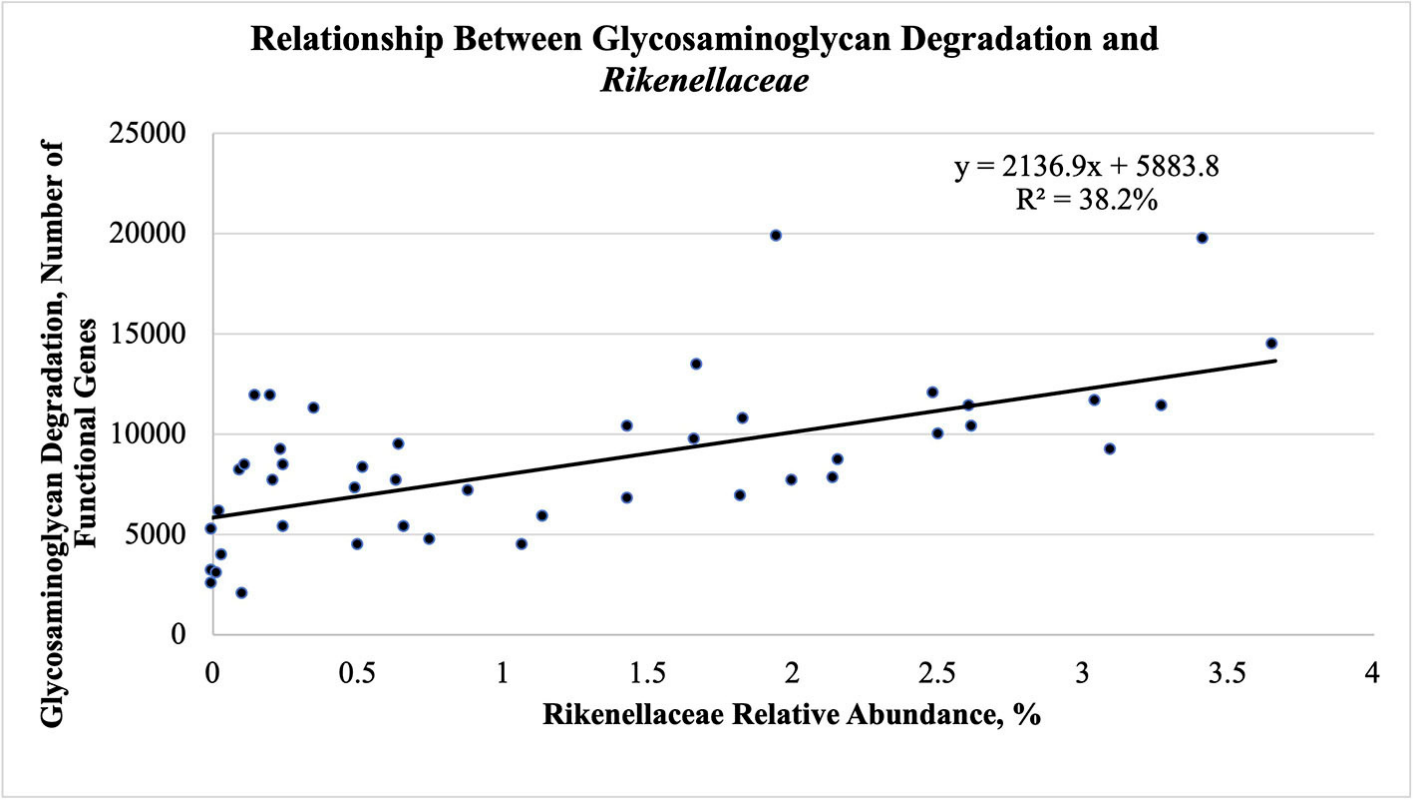
Linear regression expressing the relationship between glycosaminoglycan degradation and *Rikenellaceae* abundance in the feces of Angus steers (*n* = 15) across all stages of production.

The fecal abundance of *Rikenellaceae* was higher at weaning than at yearling and slaughter for both groups of steers (Figure 5B; *P* < 0.001). Fecal abundance of *Rikenellaceae* tended to be higher (*P* = 0.10) at weaning in the efficient steers than in the inefficient steers. At yearling and slaughter, the most efficient steers had greater fecal abundances of *Rikenellaceae* compared to the least efficient steers (*P* ≤ 0.05). As the relative abundance of *Rikenellaceae* increased in the feces during all stages of production, the expression of the gene responsible for glycosaminoglycan degradation increased (Figure 6; *r* = 0.618; *P* < 0.001; *R*^2^ = 38.2%). For both groups of steers, the abundance of *Christensenellaceae* was greater (Figure 5C; *P* < 0.001) in feces collected at weaning than in the feces collected at yearling and slaughter. Moreover, abundance of *Christensenellaceae* tended to be higher at weaning (*P* = 0.08) in efficient steers; and was higher (*P* ≤ 0.05) at both yearling and slaughter in the efficient steers.

Fecal *Bifidobacteriaceae* abundance was higher (*P* = 0.011) at slaughter compared to weaning in the inefficient steers (Figure 5D). Conversely, abundances of *Bifidobacteriaceae* remained relatively consistent throughout the life of efficient steers (*P* = 0.142), although a numerical increase was observed. At weaning, there was a greater population of *Bifidobacteriaceae* present in the feces of inefficient steers compared to the feces of efficient steers (*P* = 0.02). *Bifidobacteriaceae* abundance was on average the same in the feces of the steers at the yearling stage regardless of their feed efficiency status (*P* = 0.14). Inefficient steers tended to have greater abundances of *Bifidobacteriaceae* in the feces at slaughter compared to efficient steers (*P* = 0.06).

### Volatile Fatty Acid Concentrations

Fecal acetate concentrations were greater (Table 3; *P* = 0.002) at slaughter compared to weaning and yearling age in the inefficient steers, whereas, it was only greater (*P* = 0.019) at slaughter compared to the yearling stage in the efficient steers. Fecal acetate concentrations were greater (*P* = 0.028) in the inefficient steers compared to efficient steers at slaughter. More propionate and butyrate were present in the feces at slaughter compared to the feces at weaning and yearling (*P* ≤ 0.003) in both efficient and inefficient steers. Valerate concentrations in the feces at slaughter were higher (*P* < 0.001) in the efficient steers compared to at the weaning and yearling age; however, fecal valerate was only higher (*P* = 0.049) at slaughter compared to the yearling stage in the inefficient steers. Total VFA concentrations were increased (*P* < 0.001) in all steers, regardless of their feed efficiency, in the fecal samples collected at slaughter compared to either the weaning or yearling stages. Regardless of feedlot feed efficiency status, the ratio of acetate to propionate decreased throughout the life of the steers, being highest in the feces at weaning, intermediate in the feces at the yearling stage, and lowest in the feces at slaughter (*P* < 0.001). Correlations between VFA concentration and RFI at weaning, yearling, and slaughter can be found in Supplementary Table 3.

**Table 3:** Volatile fatty acid (VFA) concentration (m*M*) in the feces of efficient and inefficient steers at different stages of production: weaning, yearling, and kill floor (slaughter).

**Volatile fatty acid**		**Weaning**	**Yearling**	**Slaughter**	**SEM**	*P*-value^*^
Acetate	Inefficient	39.9^b^	29.6^b^	55.9^a^	4.08	0.002
	Efficient	33.0^ab^	30.6^b^	43.8^a^	2.95	0.019
	SEM	3.45	2.65	2.85		
	*P*-value	0.34	0.85	0.03		
Propionate	Inefficient	6.2^b^	6.4^b^	16.0^a^	1.05	<0.001
	Efficient	6.0^b^	5.9^b^	14.1^a^	0.82	<0.001
	SEM	0.692	0.71	1.09		
	*P*-value	0.91	0.73	0.41		
Butyrate	Inefficient	2.5^b^	4.0^b^	7.6^a^	0.87	0.003
	Efficient	1.8^b^	3.4^b^	6.5^a^	0.61	<0.001
	SEM	0.302	0.507	0.891		
	*P*-value	0.21	0.55	0.56		
Valerate	Inefficient	0.70^ab^	0.40^b^	1.09^a^	0.18	0.049
	Efficient	0.5^b^	0.3^b^	1.4^a^	0.10	<0.001
	SEM	0.098	0.137	0.182		
	*P*-value	0.31	0.83	0.48		
Total VFA	Inefficient	51.0^b^	40.4^b^	82.6^a^	5.37	<0.001
	Efficient	42.7^b^	40.2^b^	68.7^a^	3.54	<0.001
	SEM	4.52	3.78	4.08		
	*P*-value	0.38	0.98	0.09		
Acetate:Propionate	Inefficient	6.5^a^	4.6^b^	3.6^c^	0.23	<0.001
	Efficient	6.3^a^	5.2^b^	3.2^c^	0.28	<0.001
	SEM	0.202	0.189	0.200		
	*P*-value	0.54	0.05	0.37		

* *P-value for the repeated measures ANOVA using collection time as a factor*.

abc *Values not sharing a common superscript within each row are significantly different (P ≤ 0.05)*.

### Bacterial Family and Volatile Fatty Acid Correlations

At weaning, the bacterial family *Christensenellaceae* was positively correlated with propionate concentration (Table 4; *r* = 0.523; *P* = 0.046). At yearling age, the acetate to propionate ratio was positively correlated with both *Ruminococcaceae* (*r* = 0.579; *P* = 0.030) and *Rikenellaceae* (*r* = 0.675; *P* = 0.008) abundances. *Ruminococcaceae* abundance was negatively correlated to acetate (*r* = −0.578; *P* = 0.024) and total VFA (*r* = −0.536; *P* = 0.039) concentration of the feces at slaughter. This bacterial family was positively correlated to valerate concentration at slaughter (*r* = 0.515; *P* = 0.049).

**Table 4:** Correlation between volatile fatty acids concentration and bacterial families^*^ in the feces of steers (*n* = 15) at weaning, yearling, and slaughter.

	**Correlation coefficient**	***P-*value**
**Weaning**		
*Christensenellaceae*		
Propionate	0.523	0.046
**Yearling**		
*Ruminococcaceae*		
Acetate:Propionate	0.579	0.030
*Rikenellaceae*		
Acetate:Propionate	0.675	0.008
**Slaughter**		
*Ruminococcaceae*		
Acetate	−0.578	0.024
Valerate	0.515	0.049
Total VFA	−0.536	0.039

* *Only bacterial families with significance to host efficiency and significant Pearson correlations (P ≤ 0.05) with volatile fatty acids are shown*.

## Discussion

### Animal Performance

As expected, based on previous generations' performance within this commercial Angus herd (20), the steers maintained similar body weights throughout production regardless of feed efficiency classification. However, steers differed in the amount of feed consumed and in their conversion of feed into body weight. During the feedlot-finishing phase, the efficient steers consumed on average 3.9 kg less feed per day (dry matter basis) while gaining approximately the same amount of weight as their counterparts. This translated into the efficient steers needing 5.3 kg less feed than their inefficient counterparts to gain 1 kg of body weight during the feedlot trial, given that their feed:gain ratios were 10.54 and 15.84, respectively. Similarly, the calculated RFI values were distinct between the 2 groups of steers, with the lowest RFI values observed in the efficient steers. Feed conversion differences are important for producers because feed is the most expensive input cost of animal production systems, therefore having cattle that can gain the same amount of weight while consuming less feed can have a significant impact on feeder profit margins (1, 21). For instance, in the present study, it cost US$0.91 less per day to feed the more efficient steers compared to the inefficient ones, resulting in a difference of US$100.10 per steer during the 110-day feedlot trial.

### Diversity Indices

With 19% of the variation in RFI being attributed to diet composition and the digestibility of feed (5), our beta diversity results are to be expected. Since the steers in our study were transitioning from a forge-based diet at weaning to a grain-based diet at both yearling age and slaughter, it is reasonable that the differences in diets would drive the differences seen in beta diversity. The selection pressure placed on the fecal microbiota by the nutrient availability of diet selected for or against certain bacterial species causing the clustering of samples on a forage-based vs. a grain-based rations. This finding was corroborated by previous studies that found that both diet composition and age played important roles in the composition of the microbial population of the gastrointestinal tract (22, 23). So, with the age and diet of the steers changing, the microbial population within the feces is also expected to change.

Research has broadly demonstrated that more efficient ruminants have comparatively low bacterial richness and diversity (9, 24). However, studies investigating the composition of the microbiome relative to efficiency of the host mainly focus on the ruminal microbial population, so the intestinal microbiome has not been extensively evaluated in this regard (25). Shabat et al. (9) hypothesized that ruminal microbial populations with less richness and diversity carried out fewer, but more relevant metabolic pathways, leading to a more limited metabolite pool but with greater biological relevance in the rumen. However, in the intestinal environment, Welch et al. (11) found that bacterial richness and diversity were greater in both the cecal contents and feces of steers with higher feed efficiency. Other researchers have also shown that microbial diversity measures differed between the rumen and the feces of cattle (10, 18, 26). The present study found bacterial evenness and diversity to be greater in the feces of the most efficient steers which somewhat contradicts the hypothesis proposed by Shabat et al. (9); however, our findings conform the biological theory regarding the intestinal environment outlined by Welch et al. (11). Nutrient availability differs widely throughout the GIT of cattle, and the digesta that reaches the large intestine contains less-digestible nutrients, which are essentially non-digested nutrients that escaped ruminal microbial degradation and small intestinal digestion. Therefore, increased bacterial evenness and diversity in the hindgut will, in turn, result in a greater array of microbial enzymes to degrade the intestinal digesta, allowing the more efficient steers to capture more non-digested nutrients that would have been unutilized in the GIT, and to convert them into metabolic end products that can be utilized by the host.

Beyond gastrointestinal anatomy and physiology there are many other contributing factors to microbial consortium composition variation, including animal age and diet composition (10, 22, 23). Thus, the changes in alpha diversity in the present study were also related to both changes in age of the steers and dietary changes during each growth phase. Overall, we found that steers had the greatest microbial richness, evenness, and diversity in their feces at weaning, when they were younger and were reared on their dams in a pasture-based production system. During backgrounding, leading to the yearling stage, starch concentrations in their ration increased resulting in a selective pressure on the complex microbial ecosystem of their GIT. For instance, the availability of digestive enzymes (e.g., pancreatic amylase and maltase) can limit the breakdown of starch in the small intestine; therefore, starch that escapes microbial degradation in the rumen and is not digested in the small intestine, can reach the cecum and colon of cattle and impact the composition of the bacterial population colonizing them (27–29). Since the finishing feedlot ration contains an even higher concentration of starch, the selective pressure of this ration decreased the microbial evenness and diversity of all steers regardless of feed efficiency status and decreased bacterial richness (Chao1) in the inefficient steers. It has been found that as grain levels in diets increased to the point of incurring ruminal acidosis, the environmental pressures would select for reduced bacterial diversity and for a microbial population largely made up of lactic acid bacteria in the rumen (30–32). The present results tend to support this hypothesis as to a cause and type of change in the microbial consortium diversity in the feces of cattle.

### Bacterial Relative Abundances

Diet composition alters not only the diversity of the gastrointestinal microbiome as a whole, but also causes fluctuations in many individual bacterial populations within the gastrointestinal tract (33, 34). Since the steers utilized in this study changed from a pasture to a feedlot-based system, the variation in bacterial populations at each timepoint can be explained by diet. However, the more novel results were that despite fluctuations in abundance throughout the steers' lives, certain bacterial families were consistently more abundant in one group of steers based on their efficiency status, regardless of the diet.

*Ruminococcaceae* is a family comprised of primarily cellulolytic and hemicellulolytic bacterial species that produce acetate, formate, and hydrogen as fermentation end products (35, 36). This acetate production could be driving the correlation of this bacteria with the acetate-to-propionate ratio observed during the study. *Ruminococcaceae* can degrade many substrates that other bacterial families cannot because it possesses many genes which allow them to bind to cellulose, hemicellulose, and xylan, allowing them to degrade plant materials more effectively (36–39). In the present study, regardless of feed efficiency status, abundances of *Ruminococcaceae* were greatest at weaning, while animals were consuming a forage-based diet, which contains substantially more fiber than feedlot finishing diets. Genetic diversity of CAZymes (carbohydrate active enzymes) provides *Ruminococcaceae* an advantage when it comes to nutrient uptake and utilization of diverse polysaccharides (40). The negative relationship between RFI and *Ruminococcaceae* found in the present study at slaughter corroborate with our previous findings (23) that have shown that as this bacterial family increased, RFI decreased resulting in the steer becoming more efficient by utilizing a broader nutrient spectrum, allowing the animal to absorb more energy from the diet. Therefore, we suggest in the present study that the greater abundance of *Ruminococcaceae* in the most efficient steers allowed them to extract greater amounts of energy from the digesta reaching their hindguts, resulting in greater metabolizable energy levels compared to the inefficient steers.

*Rikenellaceae* is a family consisting of bacteria found within the gastrointestinal tract and fecal material from animals and humans (41, 42). Bacteria within *Rikenellaceae* can utilize mucin as a source of carbohydrates and energy which provides them a competitive advantage over other bacteria (43). This is supported by the positive relationship seen between the relative abundance of *Rikenellaceae* and the expression of the gene responsible for glycosaminoglycan degradation shown in the present study. Glycosaminoglycans are essential to the development of gastrointestinal mucosa (44). *Rikenellaceae* abundance and the gene responsible for glycosaminoglycan degradation have previously been found to be important in feces of mice in terms of gut mucosa; however, there were no correlations provided to show if a relation existed between them (45). Although the amount of glycosaminoglycans was not quantified in the present study, the positive association between *Rikenellaceae* and this gene suggests that the more efficient steers had more glycosaminoglycans present in their hindgut that can be utilized by this bacterium. *Rikenellaceae* produce acetate, succinate, and propionate as fermentative end products (46), all of which can be utilized by the host animal. This bacterial family was found to be correlated with the acetate-to-propionate ratio at yearling which can be a result of an increase in its ability to produce acetate for the host. Moreover, *Rikenellaceae* was found to be more prevalent as a member of the core microbiome of heifers fed a forage-based diet, compared to a forage-grain mixed or all grain diet (47), which agrees with present results, given that the greatest abundances of *Rikenellaceae* were observed at weaning when the steers were consuming primarily forages. The abundance of *Rikenellaceae* was consistently higher in the feces of the most efficient steers at all timepoints evaluated, indicating that the efficient steers might have produced more mucin in their hindguts resulting in a greater population of *Rikenellaceae*. Furthermore, because the abundance of this bacterial family was consistently higher in the feces of the most efficient steers, this family is a candidate marker of cattle feed efficiency, even at early stages of growth.

*Christensenellaceae* has been associated with a healthy digestive system in humans, a reduction in adipose tissue, and a lower body mass index (48). The family *Christensenellaceae* consists of species which produce α-arabinosidase, β-galactosidase, and β-glucosidase (49), which break down components of plant fibers, thus it is logical that the highest fecal abundances were found at weaning when the steers were grazing forages. Additionally, a positive correlation was observed between *Christensenellaceae* abundance and propionate concentration at weaning when this bacterial family was able to efficiently extract energy from the feedstuffs. The decrease in abundance of *Christensenellaceae* seen in the feces at yearling and slaughter can be attributed to a decrease in the overall health of the digestive system during the feedlot-finishing period (50–52), where the diet is much more concentrate-based and has been linked to a reduction in ruminal and intestinal health (53–56). Additionally, *Christensenellaceae* produce butyrate (57) which is used by the epithelial tissue for energy (58, 59). Increased butyrate production by *Christensenellaceae* may reduce the incidence of leaky gut which may be responsible for leakage of lipopolysaccharide (LPS) and resultant inflammation in the less efficient steers (55, 60–62).

*Bifidobacteriaceae* is comprised of a fructo- and galacto-oligosaccharide-fermenting bacteria to produce acetate (49). It is often associated with a healthy gastrointestinal tract and is often used as a probiotic (63–65), and has been found in the rumen, small intestine, and hindgut of ruminants (49, 66, 67). *Bifidobacteriaceae* were negatively correlated with *Christensenellaceae* populations in human fecal samples (48) which is in line with what we found in the present study. *Christensenellaceae* abundance was higher in the most efficient steers, whereas *Bifidobacteriaceae* abundance was higher in the inefficient steers. *Bifidobacteriaceae* was most abundant in the cecum of steers that had a high rate of gain and high feed intake but was not found in steers with high gain and low feed intake [i.e., more efficient; (68)]. Our present results support this finding since *Bifidobacteriaceae* populations were consistently lower in the more efficient steers.

### Volatile Fatty Acid Production

Previous studies have found low-RFI (more efficient) steers to have an increase in energy converted in the rumen (2, 59), suggesting that host efficiency may be directly related to additional VFA produced by microbial fermentation of the rumen. Welch et al. (11) reported a numerical increase in VFA production in the rumen of efficient steers compared to inefficient steers; however, fecal samples collected post slaughter were reversed, with VFA concentrations being highest in the less efficient steers. The present study observed similar patterns, with the inefficient steers having more total fecal VFA than the efficient steers. Moreover, the lower concentration of acetate and lower numerical concentrations of other major VFAs such as propionate, and butyrate observed in the feces of the efficient steers suggests that the VFA values quantified in the fecal material are strong indicators of increased lower gut VFA absorption rather than production. This result suggests a need to investigate the linkage between the microbiome of the lower gut and gut epithelial integrity and health, potentially explaining differences in feed efficiency.

Diet composition greatly impacts VFA concentration (69, 70), and it is unsurprising that fecal VFA concentrations varied greatly throughout the steers lifetimes due to the different diets consumed at each stage of growth. Furthermore, most individual VFA (and total VFA) fecal concentrations were highest at slaughter, suggesting that the hindgut microbial activity increased with age, resulting in more VFA production. The acetate-to-propionate ratio in the rumen is directly related to energy availability to the host animal because propionate is glucogenic (71–73). In the present study, the ratio of acetate-to-propionate decreased throughout the life of the steers, regardless of their efficiency status, reflecting the dietary composition as a key factor in these changes. The acetate-to-propionate ratio was decreased in ruminants fed a high concentrate diet (reflective of an increase in propionate production) compared to cattle fed a high forage diet (27, 74, 75). Although our measurements were made in the fecal material, still, the acetate-to-propionate ratio was highest when the steers consumed predominantly pasture and was lower when the steers were fed a feedlot ration.

The results presented here provide a meaningful insight into the relationship between beef steers and their fecal microbiotas throughout their productive lives. While these results are meaningful, they are reflective of a limited sample size (i.e., fifteen steers). Additionally, the samples at slaughter were collected post-mortem and following a 24-h fasting period. Thus, it is possible that these conditions impacted the fecal microbiome and VFA concentrations assessed at slaughter. Lastly, the fact that the present study identified bacteria at the family taxonomic level may pose another limitation, as more specific taxonomic levels may be more informative. Therefore, despite these notable results, more research is necessary to draw irrefutable conclusions about the relationship between beef cattle and their fecal microbiota, and how it affects their feed efficiency.

### Conclusions

Our results demonstrated that the fecal microbiota fluctuated throughout the life of beef steers, and that some specific bacterial families were consistently found at differential abundances in steers depending on their feed efficiency status. Surprisingly, for some bacterial families this holds true throughout the entire production continuum of beef steers, even when major diet changes occur. Abundances of *Ruminococcaceae, Rikenellaceae*, and *Christensenellaceae* were numerically greater in the feces of the steers with greater feed efficiency from weaning until slaughter. Conversely, *Bifidobacteriaceae* was more abundant in the feces of the less efficient steers at multiple stages of their lives, suggesting a potential negative impact on feed efficiency. Moreover, microbial diversity in the hindgut was strongly correlated with feedlot RFI, and it was consistently higher in the most efficient steers during their productive lives. Collectively, our results illustrate that the ruminants' intestinal microbiota can significantly impact feed efficiency, and some aspects of this microbiome divergence can be detected as early as weaning, leading to the opportunity for producers to utilize fecal samples as a selection tool for feed efficiency within their herd.

## Data Availability Statement

The datasets presented in this study can be found in online repositories. The names of the repository/repositories and accession number(s) can be found below: MG-RAST (accession number: mgm4909317.3).

## Ethics Statement

The animal study was reviewed and approved by approved by the University of Georgia's Animal Care and Use Committee (AUP #A2012 11-006-R1).

## Author Contributions

CW wrote the manuscript with the help of all the authors. CW, JL, TK, and DS performed data analysis. JL, TK, DS, FF, TP, and TC revised the manuscript. All authors read and approved the final version for publication.

## Conflict of Interest

The authors declare that this study received funding from Brasstown Beef LLC. The funder was not involved in the study design, collection, analysis, interpretation of data, the writing of this article or the decision to submit it for publication. Brasstown Beef LLC graciously provided intake monitored pens and labor during the study.
