# Supplementary material for: Evaluation of the Fecal Bacterial Communities of Angus Steers With Divergent Feed Efficiencies Across the Lifespan From Weaning to Slaughter
Source: Front Vet Sci. 2021 Jun 29;8:597405. doi: 10.3389/fvets.2021.597405 (PMC8275654; doi:10.3389/fvets.2021.597405)
Supplement: Supplementary file 1 [file Data_Sheet_1.PDF]

Supplementary Table 1. Composition of the diets used during the transition and finishing periods of the feedlot steers.

	Transition Diet	Finishing Diet
**Ingredient, % DM**		
Corn	41.12	56.20
Dried distillers grains	22.18	19.54
Corn gluten feed	-	7.08
Soybean Hulls	15.80	-
Barley Straw	6.15	4.36
Vitamin/Mineral Premix	4.47	4.76
Corn Silage	10.27	8.05
**Total**	**100.00**	**100.00**
**Nutrient, % DM**		
Dry Matter, %	62.00	62.00
NE_m, Mcal/kg	2.02	2.10
NE_g, Mcal/kg	1.37	1.43
Crude Protein, %	14.63	14.51
Roughage, %	16.43	12.40
Rough NDF, %	9.28	6.92
Fat, %	5.11	5.28
Calcium, %	0.75	0.70
Phosphorus, %	0.39	0.45
Potassium, %	0.90	0.71
Magnesium, %	0.22	0.21
Sulfur, %	0.25	0.26
Added Salt, %	0.21	0.22

Supplemental Table 2. Correlation between the number of genes expressed in selected bacterial metabolic pathways and bacterial families* in the feces of steers (n = 15) throughout their productive lives.

	Correlation Coefficient	*P*-value
Ruminococcaceae		
Ion channels	-0.686	< 0.001
Rikenellaceae		
Biosynthesis of 12-, 14- and 16-membered macrolides	-0.610	< 0.001
Biosynthesis of type II polyketide backbone	-0.612	< 0.001
Dioxin degradation	-0.656	< 0.001
Ether lipid metabolism	-0.635	< 0.001
Fatty acid elongation in mitochondria	0.656	< 0.001
Fluorobenzoate degradation	0.662	< 0.001
Glycosaminoglycan degradation	0.618	< 0.001
Glycosphingolipid biosynthesis - ganglio series	0.611	< 0.001
Isoflavonoid biosynthesis	0.614	< 0.001
Lipopolysaccharide biosynthesis	0.676	< 0.001
Steroid biosynthesis	0.689	< 0.001
Transporters	-0.606	< 0.001
Xylene degradation	-0.649	< 0.001
Christensenellaceae		
Dioxin degradation	-0.620	< 0.001
Fatty acid elongation in mitochondria	0.657	< 0.001
Fluorobenzoate degradation	0.677	< 0.001
Steroid biosynthesis	0.689	<0.001
Xylene degradation	-0.614	< 0.001

*Only bacterial families with significance to host efficiency and strong significant Pearson correlations ($r \geq \pm 0.600$; $P \leq 0.01$) with genes in metabolic pathways are shown.

Supplemental Table 3. Correlation between RFI of steers (n = 15) and volatile fatty acid concentration at weaning, yearling, and slaughter.

Volatile Fatty Acid	Correlation Coefficient	*P*-value
Weaning		
Acetate	0.279	0.314
Propionate	0.091	0.747
Butyrate	0.306	0.268
Valerate	0.305	0.270
Total VFA	0.262	0.346
Acetate: Propionate	0.101	0.720
Yearling		
Acetate	-0.058	0.838
Propionate	0.092	0.744
Butyrate	0.09	0.750
Valerate	-0.002	0.995
Total VFA	-0.011	0.968
Acetate: Propionate	-0.558	0.038
Slaughter		
Acetate	0.501	0.057
Propionate	0.309	0.262
Butyrate	0.168	0.549
Valerate	-0.216	0.440
Total VFA	0.436	0.105
Acetate: Propionate	0.117	0.679

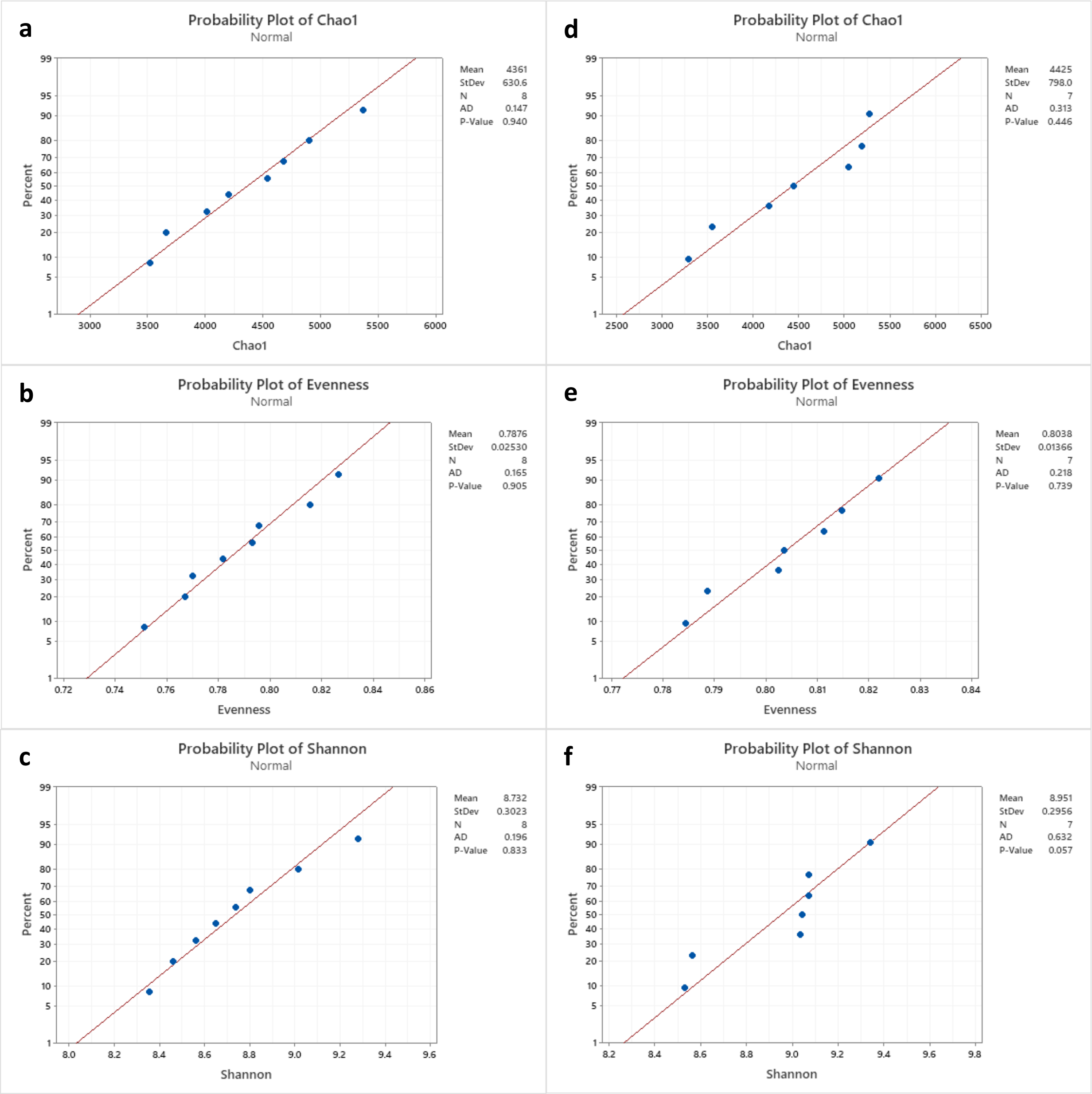

Supplemental Figure 1. Anderson-Darling Normality Tests of inefficient steers (n=8) for Chao 1 (**a**), Evenness (**b**), and Shannon Diversity (**c**) and efficient steers (n=7) for Chao 1 (**d**), Evenness (**e**), and Shannon Diversity (**f**) in feces collected at weaning.

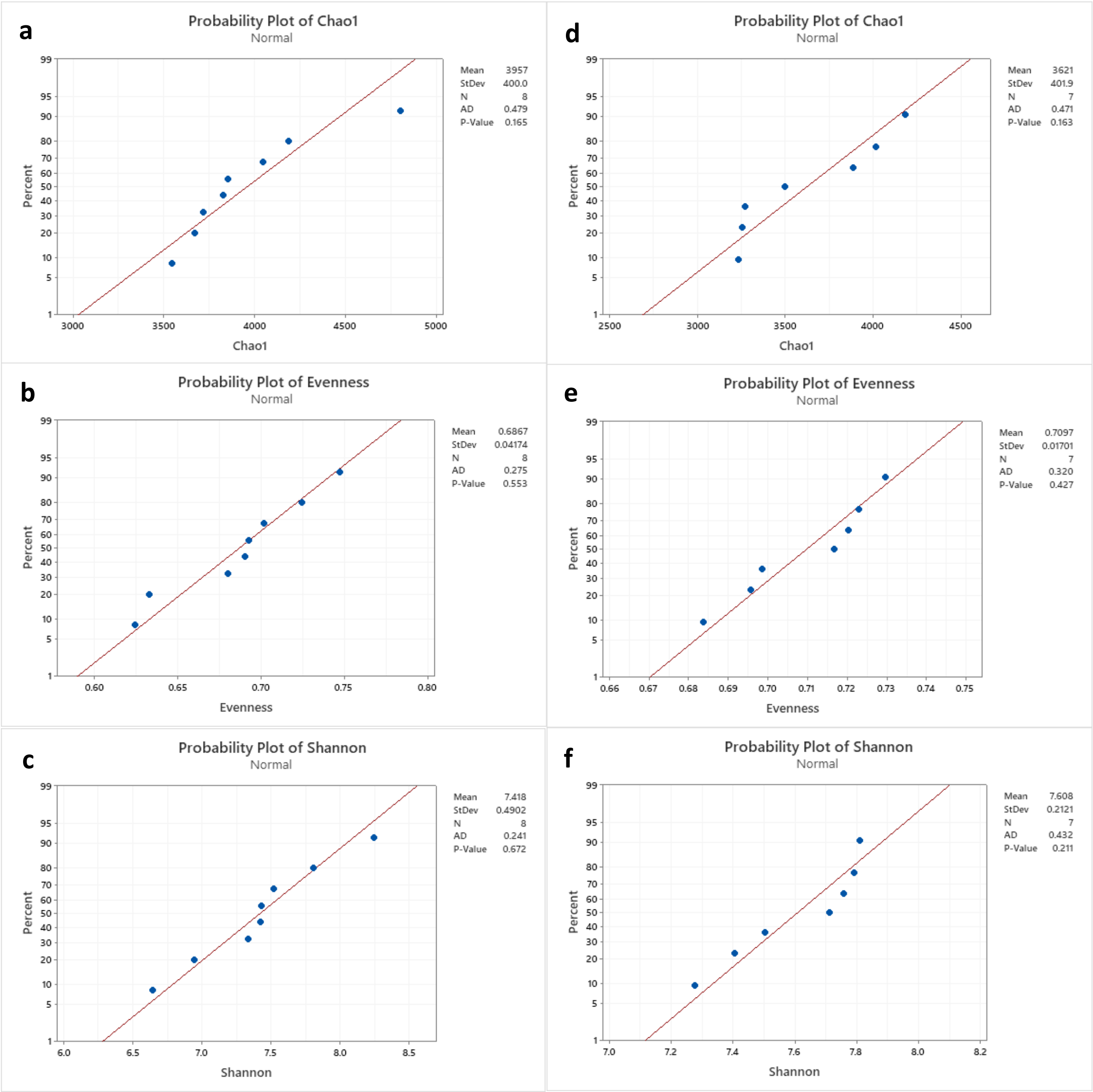

Supplemental Figure 2. Anderson-Darling Normality Tests of inefficient steers (n=8) for Chao 1 (**a**), Evenness (**b**), and Shannon Diversity (**c**) and efficient steers (n=7) for Chao 1 (**d**), Evenness (**e**), and Shannon Diversity (**f**) in feces collected at yearling.

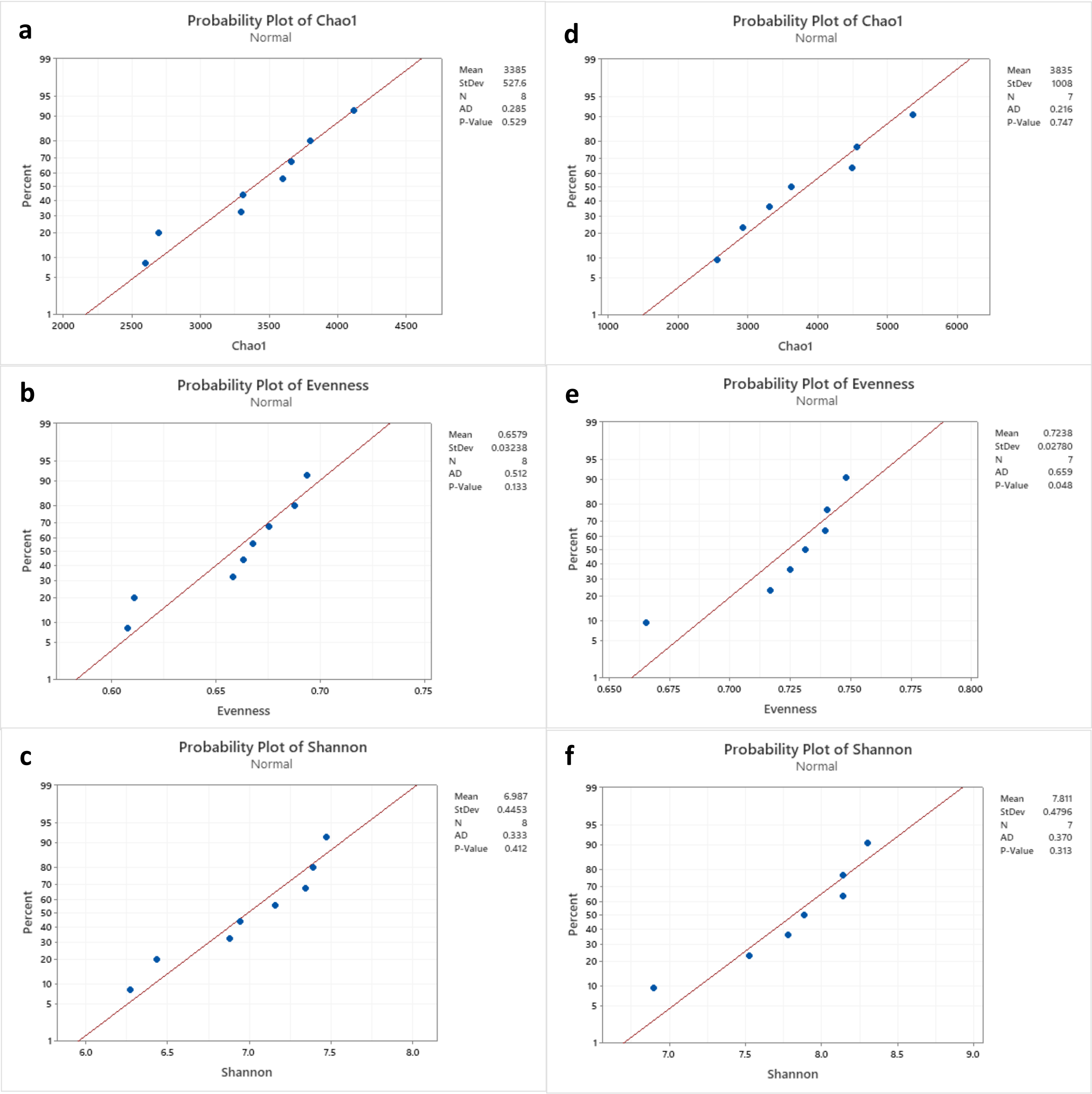

Supplemental Figure 3. Anderson-Darling Normality Tests of inefficient steers (n=8) for Chao 1 (**a**), Evenness (**b**), and Shannon Diversity (**c**) and efficient steers (n=7) for Chao 1 (**d**), Evenness (**e**), and Shannon Diversity (**f**) in feces collected at slaughter.

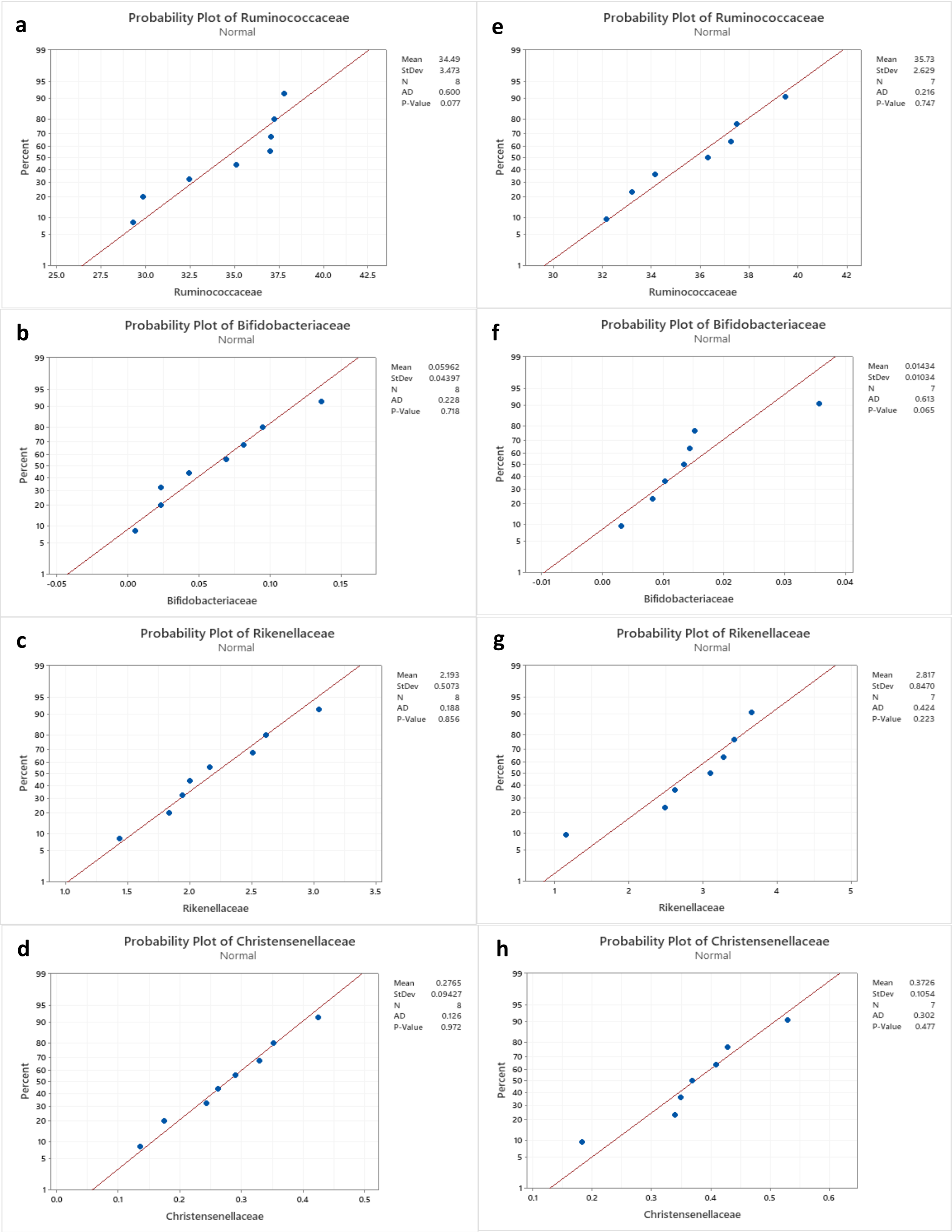

Supplemental Figure 4. Anderson-Darling Normality Tests of inefficient steers (n=8) for *Ruminococcaceae* (**a**), *Bifidobacteriaceae* (**b**), *Rikenellaceae* (**c**), and *Christensenellaceae* (**d**) and efficient steers (n=7) for *Ruminococcaceae* (**e**), *Bifidobacteriaceae* (**f**), *Rikenellaceae* (**g**), and *Christensenellaceae* (**h**) in feces collected at weaning.

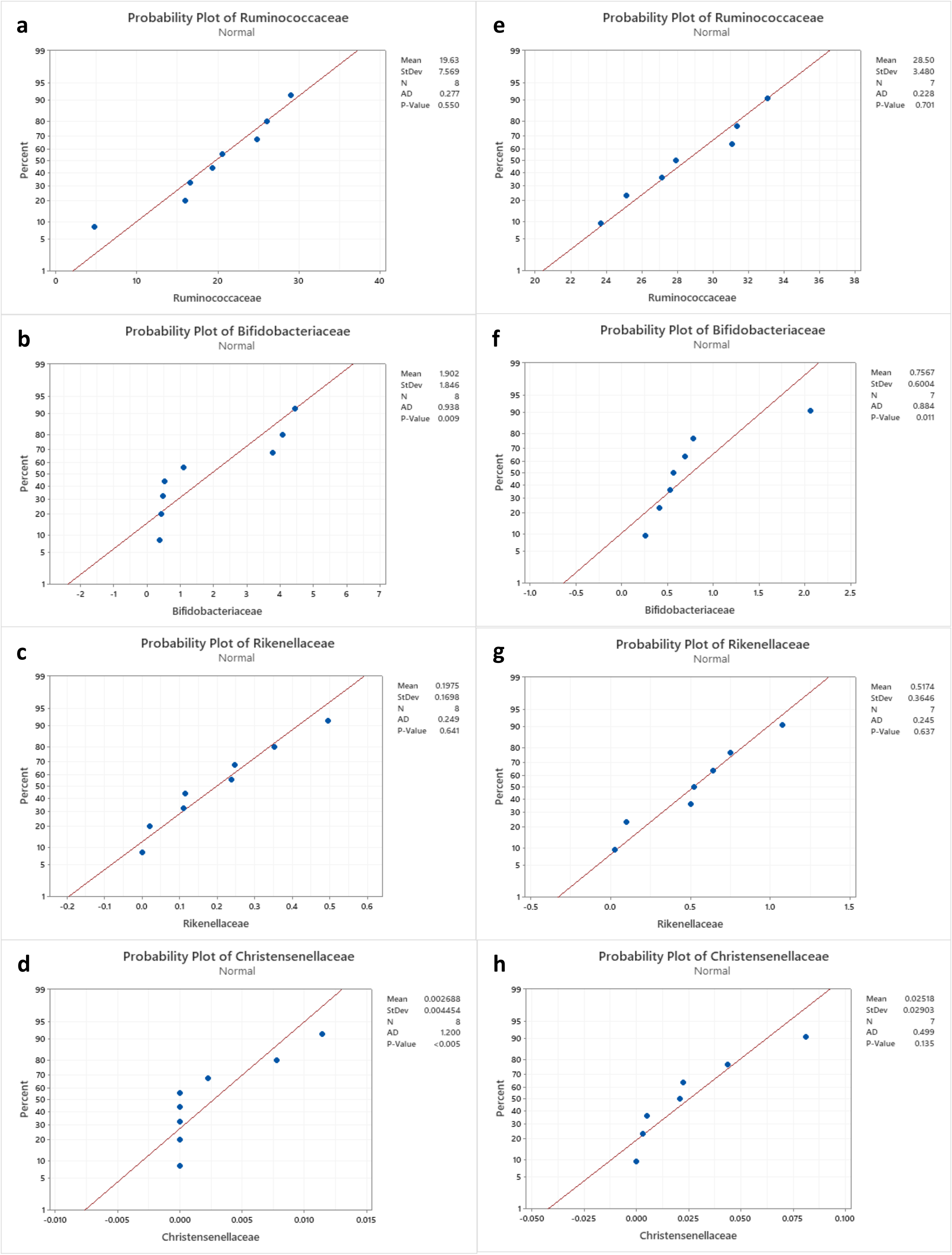

Supplemental Figure 5. Anderson-Darling Normality Tests of inefficient steers (n=8) for *Ruminococcaceae* (**a**), *Bifidobacteriaceae* (**b**), *Rikenellaceae* (**c**), and *Christensenellaceae* (**d**) and efficient steers (n=7) for *Ruminococcaceae* (**e**), *Bifidobacteriaceae* (**f**), *Rikenellaceae* (**g**), and *Christensenellaceae* (**h**) in feces collected at yearling.

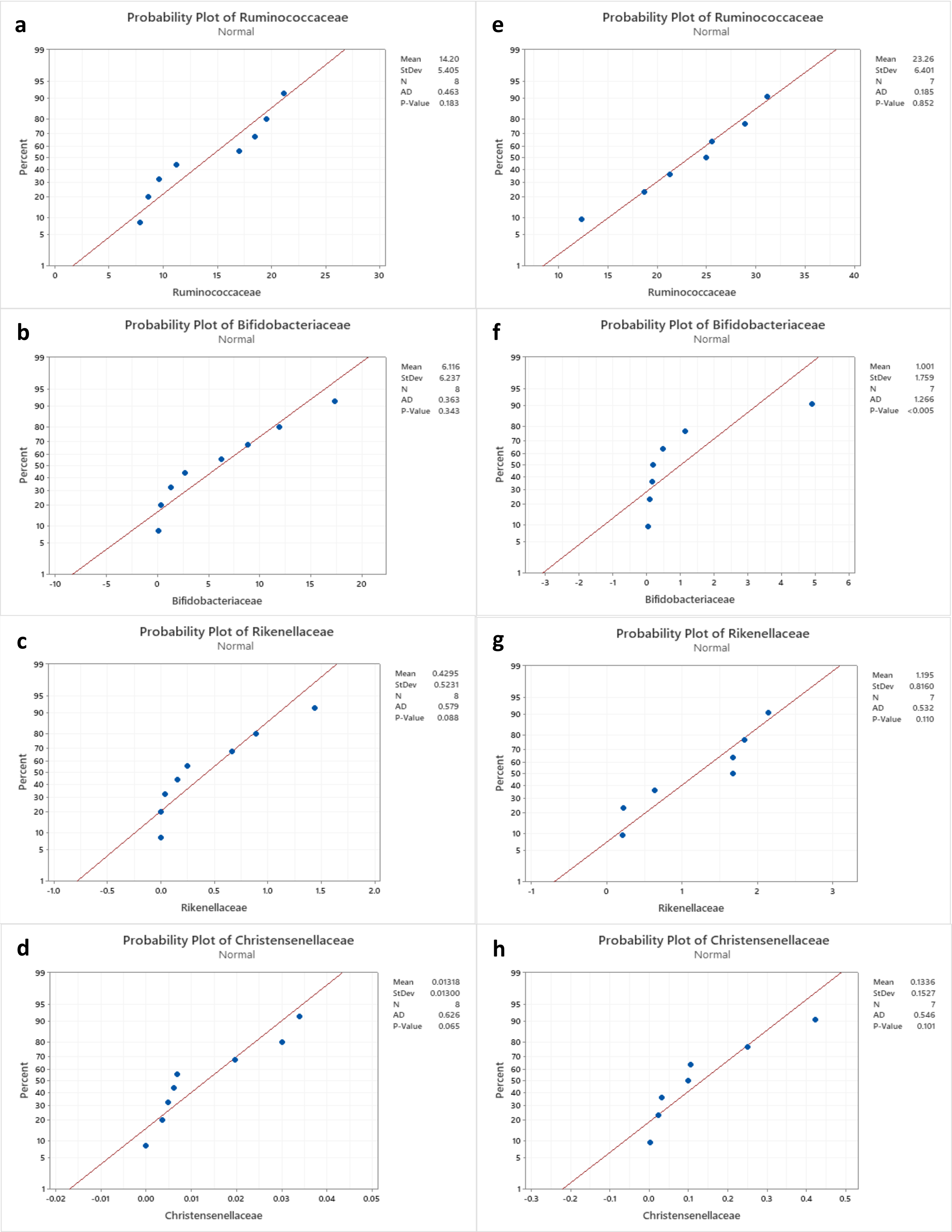

Supplemental Figure 6. Anderson-Darling Normality Tests of inefficient steers (n=8) for *Ruminococcaceae* (**a**), *Bifidobacteriaceae* (**b**), *Rikenellaceae* (**c**), and *Christensenellaceae* (**d**) and efficient steers (n=7) for *Ruminococcaceae* (**e**), *Bifidobacteriaceae* (**f**), *Rikenellaceae* (**g**), and *Christensenellaceae* (**h**) in feces collected at slaughter.

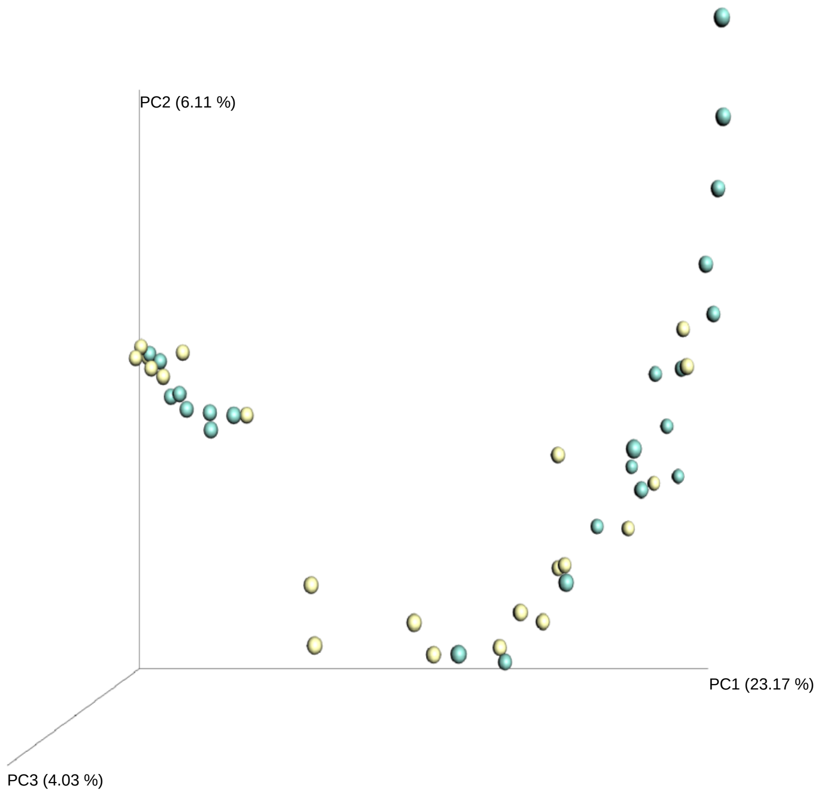

Supplemental Figure 7. Beta-diversity (unweighted UniFrac) comparing efficient (yellow) and inefficient (blue) steers. No statistical differences (P = 0.80) were detected between the two groups.
